# FULL CIRCLE: MENTORING IN GERONTOLOGY AND GERIATRICS EDUCATION

**DOI:** 10.1093/geroni/igac059.673

**Published:** 2022-12-20

**Authors:** Rona Karasik

**Affiliations:** St. Cloud State University, St. Cloud, Minnesota, United States

## Abstract

More than just a buzzword in business and education, mentoring is a complex interactional process that, at its best, fosters personal and professional development for all involved. In other words, a good mentoring relationship can be both transformative and reciprocal. This raises the question of what is (and is not) a good mentoring relationship? Moreover, how does one enter into and capitalize on the benefits of mentorship? While some mentoring relationships are intentionally created, others seem to evolve so organically that participants are not immediately aware of mentoring’s presence. The current presentation looks at the nature of mentoring relationships within the context of gerontology and geriatrics education from both the mentor and mentee perspectives.

